# Abdominal-B regulates the male seminal fluid transferome and new fecundity factors required for sperm sex peptide binding

**DOI:** 10.1371/journal.pgen.1012298

**Published:** 2026-09-01

**Authors:** Kathrin Steck, Lina Verbakel, Peter Kerwin, Vladimir Trajanovikj, Katarzyna Kjøge, Jan J. Enghild, Anne C. von Philipsborn

**Affiliations:** 1 Department for Neuroscience and Movement Science, University of Fribourg, Fribourg, Switzerland; 2 Department of Molecular Biology and Genetics, Aarhus University, Aarhus, Denmark; University of Michigan, UNITED STATES OF AMERICA

## Abstract

Seminal fluid proteins determine reproductive success in a wide range of animals. In the Drosophila male accessory gland, seminal fluid is mainly produced by two cell types, a majority of main cells and a small number of secondary cells that possess a specialized secretory apparatus with unusually enlarged dense core granule vesicles. Loss of Abdominal-B expression from secondary cells in the enhancer mutant *iab-6*^*cocu*^ disrupts their transcriptional and secretory identity. Consequently, mutant males fail to induce the long-term post-mating response in females, which is characterized by a loss of receptivity and sustained egg laying. Here, we determine how secondary cells shape the seminal transferome and the female response by assessing *iab-6*^*cocu*^ male accessory gland and female mate reproductive tract proteomes. We find downregulation of seminal fluid proteins that constitute a signaling network enabling sperm binding and the sustained action of the key regulator Sex Peptide. We identify two new Sex Peptide network proteins crucial for female fecundity, Cornutus (CG1701) and Hanrej (CG42564). Cornutus is required for mating dependent dense core granule vesicle release, providing a link between the products of these compartments and the female long-term post-mating response. Our data highlights the importance of secondary cell signaling and secretion for overall seminal fluid composition and Sex Peptide network function as well as the interdependence of main and secondary cells and their secretory products, advancing the general understanding of how seminal fluid signaling pathways modulate female physiology, sperm use and offspring production.

## Author summary

During copulation, sperm is transferred together with seminal fluid, which is crucial for protection, nutrition and modification of sperm as well as for providing signals to the female reproductive tract. A complex mixture of secreted seminal fluid proteins is required for functional seminal fluid. We use the Drosophila model to understand how different cell types in the male accessory gland, the fly’s equivalent of the prostate, cooperate in the production and transmission of seminal fluid that supports full fecundity and changes of female physiology after mating. We show that modification of the secretory identity of a small cell population in the gland, the specialized secondary cells, upon loss of expression of the transcription factor Abdominal B leads to substantial changes in the protein profile transferred to females. Among proteins downregulated, we characterize two new male factors, Cornutus and Hanrej, that are required for the binding of Sex Peptide, a key seminal fluid signal molecule, to sperm in the female storage organs and for sustained egg production and suppression of remating.

## Introduction

Seminal fluid is a key determinant for fertility and fecundity in internally fertilizing animals. It is not only a vehicle for gamete transport but also a “socially transferred material” [1] fulfilling important signaling functions that affect both sperm and the female body. Seminal fluid is produced by secretory tissues of the male reproductive tract. It contains a multitude of proteins and peptides as well as other components, including nutrients, hormones, antimicrobials, pheromones, and lipid vesicles such as exosomes. In many animals, seminal fluid proteins (SFPs) mediate sperm survival and storage, influence ovulation and fertilization, and modulate female behavior and offspring health [2–4]. In most species, the mechanisms by which SFPs exert their multifaceted effects are poorly understood and not readily accessible experimentally. *Drosophila melanogaster* has arguably the best studied seminal fluid proteome among genetic model organisms [5]. It has been instrumental for studying the effects of SFPs on females [6–8], postcopulatory sexual selection [9], the evolution of SFPs [10–13], and male allocation [14–17]. Currently, around 300 proteins are considered high confidence SFPs that are transferred from male to female. They fall into similar functional classes as the larger human seminal plasma proteome, such as proteases, protease inhibitors, redox-related proteins, proteins with immune function and proteins involved in lipid metabolism [5,18].

Despite the complex and critical roles of seminal fluid in reproductive success, only a small fraction of individual Drosophila SFPs have been functionally characterized. We lack a comprehensive understanding of the signaling pathways involved in SFP production and allocation, as well as SFP interactions with the female reproductive tract. Most research on how SFPs influence female physiology and behavior has focused on Sex Peptide (SP), which binds a neuronally expressed receptor in females and orchestrates post-mating changes in female receptivity, oogenesis and egg laying, feeding preferences and gut homeostasis, aggression and sleep (reviewed in [19–21]). SP can only exert its sustained and wide-ranging effect when bound and gradually released from sperm in the female sperm storage organs [22]. A conserved cofactor network of other SFPs is required for stable SP sperm binding and the long-term female post-mating response. The dynamics, regulation and extent of this so-called SP network are not yet fully understood [23,24].

Drosophila seminal fluid is mainly produced by the paired accessory glands (AGs). The Drosophila AG is functionally analogous to the mammalian prostate [25], with which it shares developmental programs [26], transcriptional networks [27] and growth and secretory pathways [28–30]. It consists of two secretory cell types, with around 1000 hexagonal shaped main cells and around 45 large, rounded secondary cells at the distal tip of the gland. Main and secondary cells have different transcriptional profiles. While SP is predominantly produced by main cells, expression of some SP network members (*lectin46-Ca*, *lectin46-Cb* and *CG17575)* is highly enriched in secondary cells. Secondary cells are characterized by a specialized secretory apparatus with unusually large vesicles containing exosomes and dense core granules (DCGs) [31–36]. For fully functional seminal fluid, interaction between both cell types and their products is required [37]. This is illustrated by the drastic effect of transcriptional dysregulation of secondary cells in a *Hox* gene enhancer mutant. Specific loss of Abdominal-B (Abd-B) expression in secondary cells of *iab-6*^*cocu*^ mutant males transforms their typical morphology and abolishes the large DCG vesicles. Females mated to *iab-6*^*cocu*^ mutant males fail to maintain the long-term post-mating response, show a high rate of remating and low fecundity [38,39] and do not respond with normal acoustic signals to seminal fluid in copula [40]. SP and the main known SP network proteins were still found to be present in *iab-6*^*cocu*^ mutant males [39]. Transcriptome analysis of *iab-6*^*cocu*^ mutant AGs, with functional analysis of selected candidates, indicated that most of the downregulated genes influencing the female long-term post-mating response were not SFPs but acted indirectly on seminal fluid composition by altering secondary cell physiology and secretory activity [41]. The complex phenotype of *iab-6*^*cocu*^ mutants, the contribution of secondary cell specific secretions to seminal fluid composition and a functional SP network are not yet fully understood.

To gain better insight into how the seminal fluid proteome and transferome are changed upon loss of Abd-B expression in secondary cells, we assessed protein abundances by mass spectrometry of male and mated female tissues. Among SFPs downregulated in the *iab-6*^*cocu*^ mutant transferome, we found four previously characterized SP network proteins and discovered two additional SFPs that are indispensable for the long-term post-mating response. The two new fecundity and receptivity factors, *cornutus* (CG1701) and *hanrej* (CG42564), are required in secondary and main cells respectively, to suppress female remating and sustain ovulation by mediating SP sperm binding. Cornutus is required for mating induced DCG release and affects aspects of DCG maturation. Our findings highlight how specialized secretion pathways maintained by Abd-B expression in secondary cells shape the overall transferome from the AG by broadly regulating abundances of SP network proteins.

## Results

### Seminal fluid proteome and transferome changes in *iab-6*^*cocu*^ mutant males

**Fig 1:**
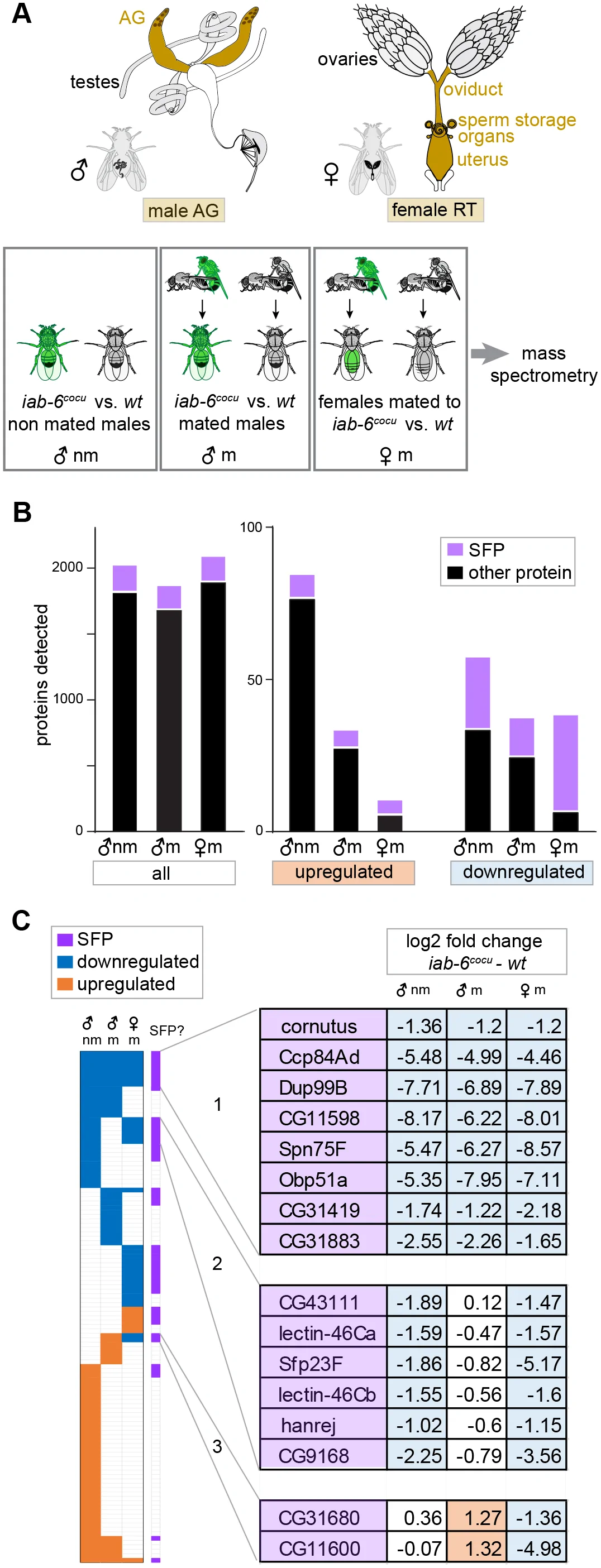
Downregulation of the seminal fluid transferome in *iab-6*^*cocu*^ mutants. **A** Experimental design of differential mass spectrometry of male and female reproductive tissues for determination of *iab-6*^*cocu*^ mutant proteome and transferome changes **B** Number of proteins detected, upregulated and downregulated in *iab-6*^*cocu*^ mutants/mutant mates vs wild type controls. The three datasets are AG proteome non mated males (♂nm), AG proteome mated males (♂m) and non mated male, and RT proteome mated female (♀m). SFPs are shown in lilac. **C** List of high confidence transferome changes, i.e., proteins downregulated in the RT proteome mated female (♀m) and consistently changed in male proteomes (♂nm and ♂m), ordered based on hierarchical clustering into three groups. Numbers give the log2 fold change of protein abundance in *iab-6*^*cocu*^ - wild type samples. Instances of log2 fold change ≤ -1 and adjusted p-value < 0.05 are indicated in blue (significantly downregulated in *iab-6*^*cocu*^ samples), instances of log2 fold change ≥1 and adjusted p-value < 0.05 (significantly upregulated in *iab-6*^*cocu*^ samples) are indicated in orange. SFPs are shown in lilac. For complete list, see S2 Table.

To understand how the seminal fluid proteome in the male accessory gland (AG) as well as the transferome reaching the female reproductive tract is changed in *iab-6*^*cocu*^ mutants, we performed liquid chromatography tandem mass spectrometry of three tissue types: virgin male accessory glands (AG proteome non mated males), accessory glands of males collected immediately after the end of copulation (AG proteome mated males) and the female reproductive tract (RT) including uterus and sperm storage organs collected immediately after copulation (RT proteome mated female). For unmated and mated AG proteome assessment, we compared *iab-6*^*cocu*^ mutants to wild type controls. For mated female RT proteome assessment, we compared wild type females mated to wild type males with wild type females mated to *iab-6*^*cocu*^ mutants (Fig 1A).

Across the three datasets, we identified 2037, 1882, and 2104 proteins with high confidence at the peptide and protein levels (1% FDR). Of the identified proteins, 85, 34 and 11 are significantly more abundant in *iab-6*^*cocu*^ samples and 58, 38 and 39 are significantly less abundant (adjusted p-value < 0.05, 2 ≥ fold change) (Fig 1B, S1 Table).

In the three sample pairs, we identified 209, 188 and 199 of a total of 292 known high-confidence SFPs [5]. While known SFPs amount to approximately 10% of the total number of identified proteins, they are significantly enriched among the differentially abundant SFPs, constituting 22% (n = 32), 26% (n = 19) and 74% (n = 37) (Odds ratios of 3.08, 3.48 and 33.2, p < 0.0001, Fisher’s exact test) of the differentially abundant proteins in the AG proteome of mated and unmated males and in the mated female RT proteome, respectively (Fig 1B, S1 Table).

Differentially abundant proteins in the mated female RT could be due either to transferome changes in *iab-6*^*cocu*^ mutant males or to differential translation/ degradation of female proteins in response to receiving a mutant *iab-6*^*cocu*^ transferome. To address transferome changes, we considered proteins detected in all three datasets (n = 1158) and focused on those that showed significant abundance changes in the mated female RT proteome and in one or both male proteomes, consistent with a transferred SFP. A single protein (Acp26Ab) is increased in all three datasets. Among proteins significantly decreased in the mated female RT proteome, we focused on three groups that also showed consistent changes in male proteomes. Group 1 proteins are significantly decreased in both male proteomes (n = 8). Group 2 proteins are decreased in the unmated male proteome and unchanged in the mated male proteome (n = 6). Group 3 proteins are unchanged in the unmated male proteome and increased in the mated male proteome (n = 2). We conclude that group 1 and 2 proteins are found in lower abundances in females mated to *iab-6*^*cocu*^ males because they are present in lower abundances in AGs of *iab-6*^*cocu*^ males. Group 3 proteins are most likely not affected in their abundances in *iab-6*^*cocu*^ males, but rather in their ability to be transferred to the female. We assume that the 16 SFPs from groups 1–3 represent direct male transferome changes rather than a differential female response and will refer to them in the following as “transferome downregulated SFPs”. All 16 proteins are high-confidence SFPs as classified by Wigby et al. [5] (Fig 1C, S2 Table). According to FlyAtlas3 Anatomy RNA-Seq data ([42], FlyBase release FB2026_02), all 16 genes are expressed in the male AG, with the majority of them showing extremely high or very high expression levels, and only moderate expression in a few other organs (S3 Table).

### SFPs depleted in the *iab-6* ^*cocu*^ mutant transferome affect female post-mating receptivity

Next, we wondered if any of the 16 transferome downregulated SFPs were implicated in phenotypes associated with *iab-6*^*cocu*^ males, namely their incapacity to trigger acoustic behavior in female mates during copulation [40] and failure to suppress female receptivity to remating [39].

We tested 15 of the 16 downregulated SFPs for which RNAi lines were available at public stock centers, using a ubiquitous driver line (*tubulin-GAL4*) for knockdown of gene expression in male flies and recorded female copulation song (S1 Fig) as well as remating rates of their wild type mates (S3 Table). Depletion of the candidate SFPs by RNAi does not cause a significant decrease in female copulation song in mates of experimentally manipulated males for any of the lines tested (S1 Fig).

Among the downregulated SFPs, Lectin-46Ca, Lectin-46Cb and CG42564 (renamed Hanrej) have been previously reported to be required for suppression of female receptivity to remating [23,24,43]. Among the remaining candidates, we find that ubiquitous knockdown of CG1701 (renamed Cornutus) leads to a large proportion of females remating at 4d after the first mating when tested with a single wild type male for 1h (S3 Table, 76%, n = 25 and 74%, n = 15, with two independent RNAi lines, respectively as compared to 0%, n = 0/30 for the genetic driver line control).

Additionally to the 16 transferome downregulated SFPs, we analyzed the list of proteins downregulated in the mated female RT proteome, which did not show consistent changes in the male proteomes (n = 12) or were not detected in both male proteomes (n = 4) but were high-confidence SFPs (S1 Table). Among these proteins, two additional mediators of the post-mating receptivity response have been reported: Aquarius [44] and CG9997 [43]. As Lectin-46Ca and Lectin-46Cb, Aquarius and CG9997 belong to a small group of so-called SP network proteins that mediate binding of SP to sperm, movement of SP into female storage organs and sustained, SP dependent post-mating responses in females [23,45]. The involvement of Hanrej/CG42564 in SP sperm binding has not been experimentally tested. Still, the protein has been suggested to be a SP network member based on evolutionary analysis of correlated gene copy variation [24].

We conclude that downregulation of four characterized SP network proteins, as well as two new receptivity affecting proteins (Cornutus and Hanrej) in the transferome of *iab-6*^*cocu*^ mutant males, is likely to contribute to increased post-mating receptivity of their female mates.

### Functional characterization of two new mediators of the long-term female post-mating response, *cornutus* (CG1701) and *hanrej (*CG42564)

Cornutus/CG1701 and Hanrej/CG42564 were found to be transferred from male to female in proteomics studies using isotope-labeled females and thus were identified as SFPs [46,47] but have not been functionally studied in detail so far.

**Fig 2:**
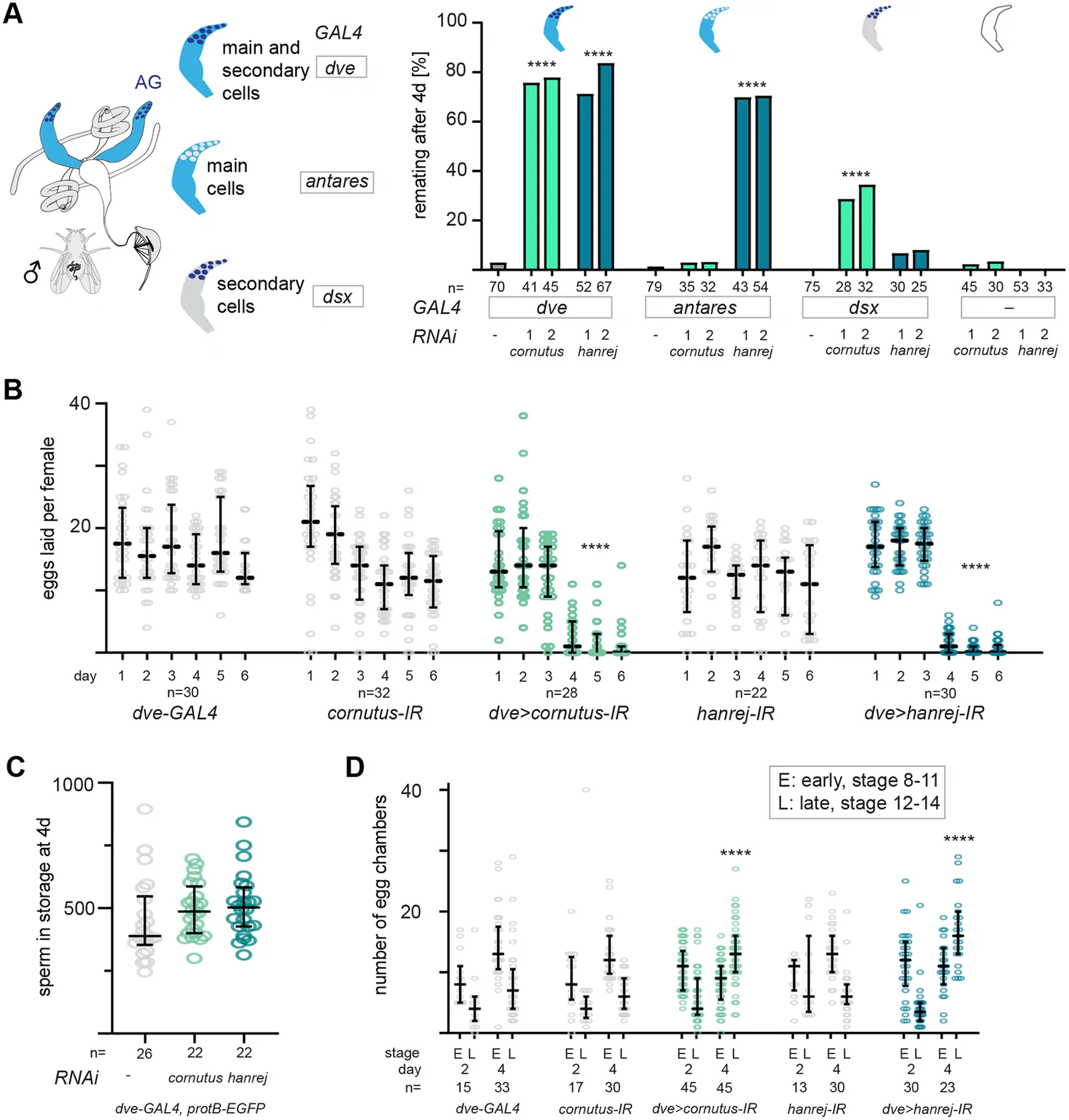
Cornutus and Hanrej are required for the sustained female post-mating response. **A** Schematic of driver lines used for expressing RNAi in different cell types of the AG (left). Percentage of remating female flies after 4d when mated to males in which *cornutus* o*r hanrej* were depleted with RNAi expressed in different AG cell populations and to control males (right). ****p < 0.0001, Fisher’s exact test. n, number of females tested, is indicated under the x axis. **B** Number of eggs laid by females mated to males in which *cornutus* o*r hanrej* were depleted and to control males, over the course of six days following the mating. ****p < 0.0001, Kruskal Wallis test with Dunn’s multiple comparison. **C** Number of sperm detected in female storage organs at 4d after mating for females mated to males in which *cornutus* o*r hanrej* were depleted with RNAi and to control males. No significant difference was detected between knockdown mates and the control (Mann-Whitney test, two sided). **D** Number of egg chambers, divided by E, early (8-11) and L, late (12-14) stages at day 2 and day 4 after mating found in females mated to males in which *cornutus* o*r hanrej* were depleted with RNAi and to control males. ****p < 0.0001, Kruskal Wallis test with Dunn’s multiple comparison. For full genotypes, see methods. Error bars represent median and interquartile range. n, number of females tested, is indicated under the x axis.

Given the strong effects on female post-mating receptivity and the phenotypic similarity of their knockdowns to the *cocu* mutant allele of *iab-6*, we propose new names for the previously unnamed genes: *cornutus* (CG1701) and *hanrej (*CG42564), the Latin and Danish equivalent of the French “cocu”, cuckold. Cornutus is a 134aa protein with no known characterized protein domain conserved within the melanogaster group. Hanrej is 500aa cysteine rich protein with a CAP domain that is orthologous to human CRISP (cysteine rich secretory protein) family proteins with reproductive function (FlyBase, release FB2026_02). Single-cell transcriptomics data indicate that expression of both genes is enriched in the AG and predominates in main cells [35,48]. To explore cell-type specific requirements, we used GAL4 driver lines labeling both main and secondary cells (*dve-GAL4*, [49]), main cells exclusively (*antares-GAL4*, a CRIMIC Trojan-Gal4 line from a gene-specific library, [50]) and secondary cells exclusively (*dsx-Gal4*, [51]) (Figs 2A, S2A) to knock down *cornutus* and *hanrej*. We confirmed that the cell type specific expression of these three drivers was consistent and persisted during 7 days in the AG of unmated males (S2B Fig). Wild type levels of *cornutus* and *hanrej* mRNA are required in secondary cells and main cells of the AG, respectively, to prevent remating of females mated to experimental males at 4d after the first mating (Fig 2 A). Significant remating also occurred already at 2d and at 3d after the first mating when *hanrej* was knocked down with drivers targeting main cells and at 3d when cornutus was knocked down with a driver targeting both main and secondary cells (S3A Fig). To confirm specificity of the phenotype to downregulation of protein in the AG, we combined the *dve-GAL4* driver, which also expresses in the innervation of the male RT, with a pan-neuronal *nsyb-GAL80*, suppressing nervous system expression. Introducing *nsyb-GAL80* led to a complete loss of nervous system GAL4 expression and slightly decreased GAL4 expression in secondary cells. With this genotype, we obtained significant remating for knockdown of *cornutus* (S2C, S2D Fig).

Concomitant with the increase of post-mating receptivity, there is a drastic drop in fecundity, measured by the number of eggs laid per female, at 4d after mating, in mates of *cornutus* and *hanrej* knockdown males with the *dve-GAL4* driver (Fig 2B). Egg laying is also strongly reduced in mates of *hanrej* knockdown males with the main cell specific *antares-GAL4* driver. Mates of *cornutus* knockdown males with the secondary cell specific *dsx-GAL4* driver do not show a significant reduction in egg laying compared *dsx-Gal4* driver control flies, which also have low fecundity after 3d (S3B Fig).

At 4d after mating, mates of knockdown males have the same number of sperm in their storage organs (seminal receptacles and spermatheca) as mates of control males (Fig 2C). In the ovaries of mates of knockdown males, an accumulation of late-stage egg chambers is seen at 4d, but not 2d after mating (Fig 2D). We do not see ovulated unlaid eggs accumulating in the oviduct, a phenomenon that has been previously observed upon blocking neuronal circuits for egg laying [52,53]. Since both sperm and mature egg are present, but no eggs are released from the ovaries, we presume that Cornutus and Hanrej are required for ovulation.

### Cornutus and Hanrej mediate binding of Sex Peptide to sperm

**Fig 3:**
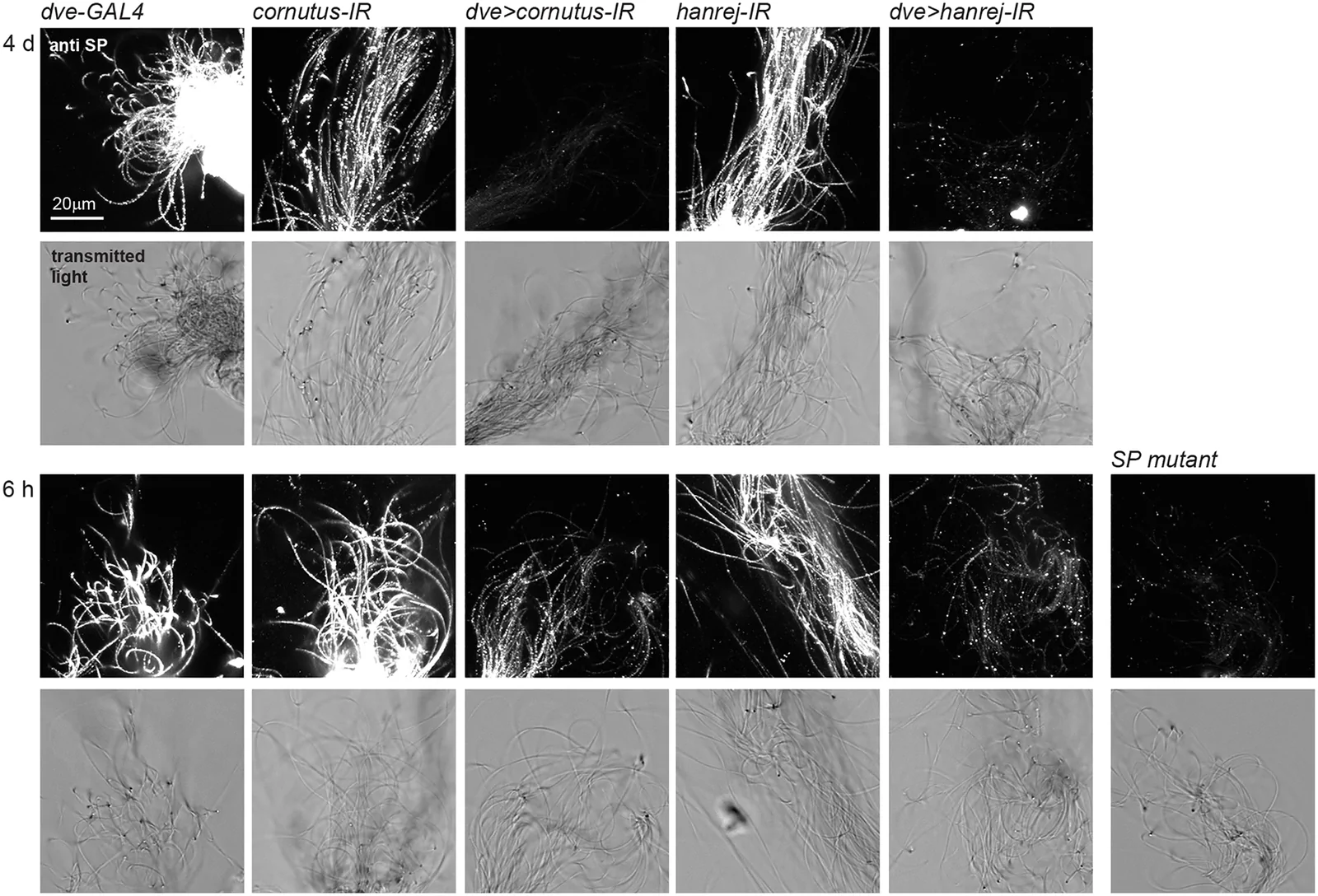
Cornutus and Hanrej are required for Sex Peptide binding to sperm. Immunohistochemistry of SP (anti SP) on sperm dissected from seminal receptacles and visualized in transmitted light 4d (above) and 6h (below) after the female’s mating to males in which *cornutus* o*r hanrej* were depleted with RNAi and to control males, as well as sperm from a female mated to a *SP* mutant male. For full genotypes, see methods. Scale bar, 20μm. A micrograph of one representative sperm bundle (out of 3-8 samples per condition) is shown.

The phenotypes observed upon *cornutus* and *hanrej* knockdown resemble closely the ones previously found for members of the SP network [23]. To test if Cornutus and Hanrej are required for the long-term association of SP with sperm, we assessed the presence of SP on sperm in the seminal receptacle by immunohistochemistry in females that were mated to males depleted for these proteins. At 4d after mating, the time point when females mated to knockdown males almost completely stop laying eggs, there is no detectable SP bound to sperm in these females, whereas females mated to control males have sperm with SP bound to its tails (Fig 3). Shortly after mating, at 6h after the end of copulation, when sperm storage is complete, sperm in seminal receptacles from males in which Cornutus or Hanrej were depleted show markedly weaker, punctate SP staining than sperm from control males (Figs 3, S4A Fig). Consistent with the findings for 4d and 6h, we also see decreased SP staining in mates of knockdown males at 2d and 3d after mating (S4B Fig).

This leads us to conclude that Cornutus and Hanrej are required for efficient binding of SP to sperm tails shortly after mating and for the long-term presence of SP in the female receptacle.

### Cornutus affects dense core granule maturation and secretion in accessory gland secondary cells

**Fig 4:**
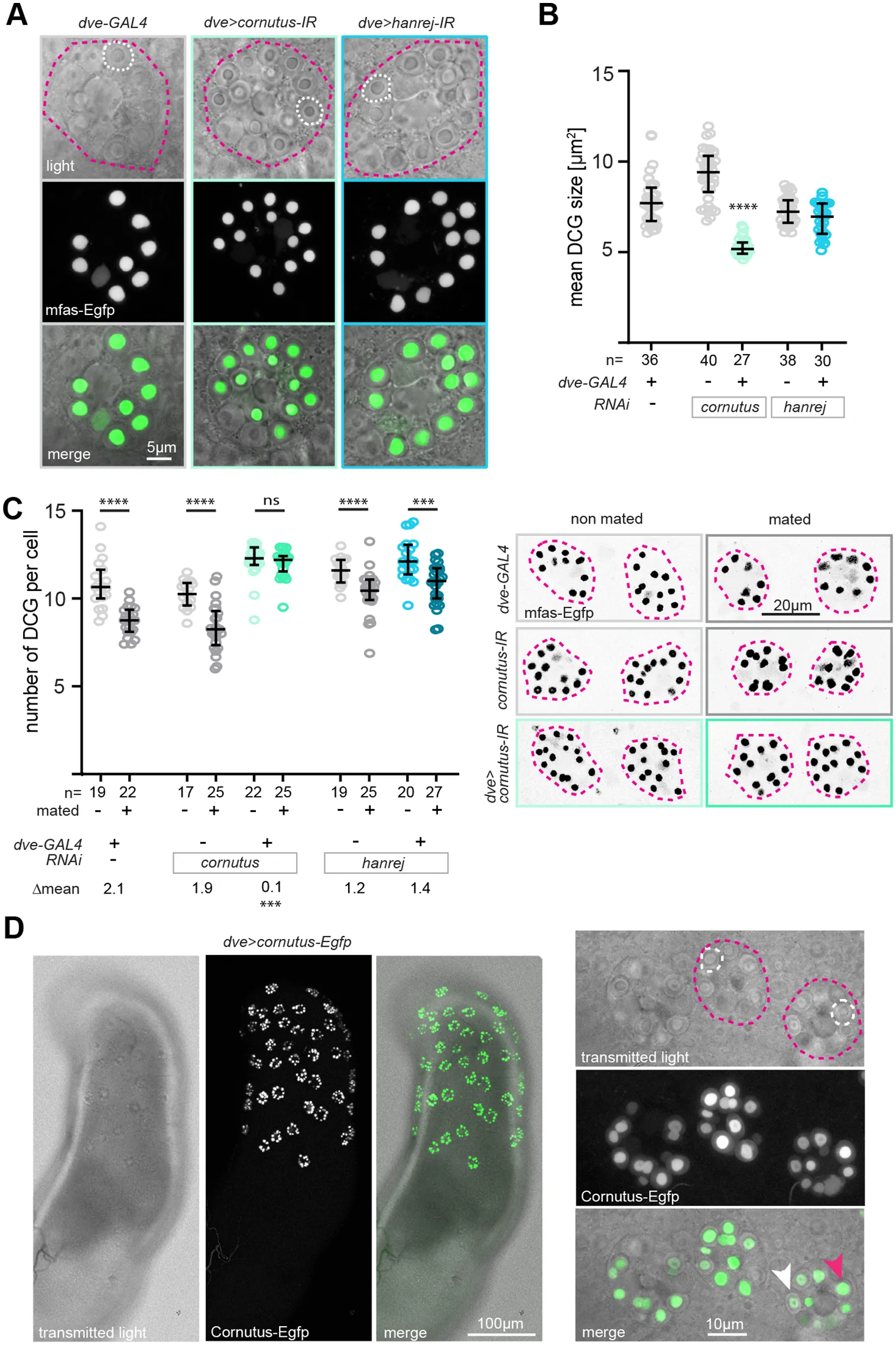
Cornutus is required for dense core granule maturation and release. **A** Representative images of secondary cells of control and knockdown males with mfas-Egfp labelled DCGs. In the transmitted light images, the circumference of the secondary cell is indicated by a pink dashed line, and one of the large secretory vesicles is outlined by a white dashed line. Scale bar, 5μm. **B** Mean size of mfas-Egfp labelled DCGs in control and knockdown males. n, number of secondary cells evaluated, is indicated under the x axis. ****p < 0.0001, Kruskal Wallis test with Dunn’s multiple comparison. **C** Mean number of DCGs per secondary cell in in control and knockdown males, for unmated and mated males. n, number of accessory glands evaluated, is indicated under the x axis. ****p < 0.0001, Mann Whitney test. The difference between unmated and mated males (Δmean) is indicated for each genotype. ***p = 0.0006, permutation testing. On the right, DCGs of two representative secondary cells (mfas-Egfp in black, outline of cells indicated by pink dashed lines) are shown for each genotype and mating state. Scale bar, 20μm. For full genotypes, see methods. Error bars in **B, C** represent median and interquartile range. **D** Localization of Cornutus-Egfp, expressed with the *dve-GAL4* driver, in secondary cells DCGs. On the left, the distal part of an accessory gland is shown. On the right, three adjacent secondary cells (two indicated by a dashed pink line, with two exemplary large secretory vesicles outlined by a dashed white line) are shown. An example of strong Cornutus-Egfp label (magenta arrowhead) and one with fainter Cornutus-Egfp label (white arrowhead) in DCGs is indicated. Scale bars, 100μm and 10μm.

In *iab-6*^*cocu*^ enhancer mutant males, the secondary cells of the AG lack large secretory vesicles (also called vacuoles) that are characteristic of this cell type [39]. We used a newly described marker for secretory vesicles, mfas-Egfp, that accumulates in DCGs [54] to assess secondary cell morphology in knockdown males. Upon knockdown of either *cornutus* or *hanrej* in the accessory gland, we still observe mfas-Egfp positive DCGs within the large secretory vesicles of secondary cells (Fig 4A). However, DCG are smaller when Cornutus is depleted (Fig 4B). Together with the decreased size of DCGs in *cornutus* knockdown males, the mean number of DCGs increases per cell (p = 0.001, Kruskal Wallis test with Dunn’s multiple comparison, comparing the mean DCG number of non-mated males, Fig 4 C). Previously, it has been shown that a small number of mature DCGs are secreted from each secondary cell upon mating [55]. In agreement with this, we see around two mfas-Egfp labelled DCGs per cell being lost during mating in control flies. Upon knockdown of *cornutus*, the mean number of DCGs per cell did not differ before and after mating (Fig 4C). Such a loss of mating dependent DCG number reduction was not seen after knockdown of *hanrej* (Fig 4C). To assess the intracellular distribution of Cornutus, we expressed C-terminally Egfp-tagged Cornutus with the *dve-GAL4* driver. The tagged protein is detected in DCGs of secondary cells (Fig 4D). In contrast to mfas-Egfp, Cornutus-Egfp does not label all DCGs with similar intensity. While some DCG are strongly and uniformly labeled, other have weaker Egfp signal that is excluded from the center of the DCG (Fig 4D). This indicates that Egfp-tagged Cornutus might accumulate in DCGs over their course of maturation. Cornutus-Egfp labels DCGs in unmated males at the day of eclosion (0d) and at 4d and 10d after eclosion. Consistent with the observation that only few DCGs per secondary cell are released during mating, Cornutus-Egfp positive DCGs are also present in mated males. In unmated males at 10d, additionally to DCG label in secondary cells, smaller Egfp labeled puncta can be seen main cells and a diffuse label in the AG lumen (S5 Fig).

In summary, these results provide evidence that Cornutus is a protein localizing to DCGs and required for proper DCG maturation and secretion in accessory gland secondary cells.

## Discussion

### Abdominal-B expression in male accessory gland secondary cells shapes the seminal fluid transferome and transfer of Sex Peptide network proteins

Female RT proteome analysis reveals that females mated to *iab-6*^*cocu*^ mutants contain lower abundances of four well characterized SP network proteins, Lectin-46Ca, Lectin-46Cb, Aquarius and CG9997. Since each SP network protein is required individually for the female long-term response, combined downregulation of these four network proteins could be sufficient to explain the *iab-6*^*cocu*^ mutant post-mating phenotype.

Overall, more than 10% of the known SFPs we detected in the AG and female RT proteome were differentially abundant in *iab-6*^*cocu*^ mutants or females mated to these mutants, respectively.

To stringently filter high confidence transferome changes in *iab-6*^*cocu*^ mutants, we focused on proteins that were consistently changed in the data sets from unmated and/or mated males as well as in mated females, obtaining a list of 16 proteins with lower abundances in the RT of females mated to mutants, all of which were SFPs. Functional testing of these transferome-downregulated SFPs revealed two new SP network proteins, CG1701/Cornutus and CG42564/Hanrej, further strengthening the indication that SP network proteins are overrepresented among the SFPs downregulated in the *iab-6*^*cocu*^ transferome.

Interestingly, none of these SP network proteins was previously found to be downregulated in *iab-6*^*cocu*^ mutants at the level of mRNA [41]. Small changes in transcription might have not been detected, and/or protein abundancy changes of SP network members might depend on a shared mechanism for secretion, or post-secretion stabilization and transport that is orchestrated by Abd-B target genes in secondary cells.

Possibly, some *iab-6*^*cocu*^ transferome changes could stem from indirect mechanisms and secondary cells, via their unique transcriptional and secretory profile, could also modulate main cell transcription, SFP abundances and transfer. For example, we find two SFPs with a main cell expression bias (CG31680 and CG11600) significantly depleted in the female, present at normal levels in unmated mutant males, and elevated in mated mutant males, a pattern that clearly suggests failed transport. Only a single protein from our downregulated transferome list, CG11598, was previously reported to be strongly transcriptionally downregulated in *iab-6*^*cocu*^ mutants [41]. Single cell transcriptomics [35] suggests that among the 16 downregulated transferome SFP genes, only two, *lectin-46Ca* and *lectin-46Cb*, exhibit a strong expression bias in secondary cells. On the other hand, four genes are significantly enriched in expression in main cells and two (*Dup99B and Obp51a*) in the anterior ejaculatory duct. The SP homologue Dup99B, though not implicated in the SP network, and not required for the post-mating response in the presence of SP [56], can induce post-mating responses [57]. In *iab-6*^*cocu*^ mutants, reduced Dup99B might contribute to defects in the post-mating response in addition to impaired SP network signaling.

Taken together, the *iab-6*^*cocu*^ transferome illustrates strong post-transcriptional control of SFP networks and interdependence of main and secondary cell products.

### Hanrej and Cornutus: New members of the Sex Peptide network ensuring the long-term female post-mating response

Among the SFPs downregulated in the *iab-6*^*cocu*^ mutant transferome, we find two new regulators of female fecundity and receptivity to remating, CG1701/Cornutus and CG42564/Hanrej. RNAi mediated knockdown of these two factors fully recapitulates the failure of the long-term post-mating response seen for *iab-6*^*cocu*^ mutants or single characterized members of the SP network. In females mated to *cornutus* or *hanrej* knockdown males, production of fertilized eggs is normal for three days, but then drops sharply, coinciding with high remating rates at four days after mating. Females normally remate and stop laying eggs when they have depleted stored sperm [58]. Females mated to *cornutus* or *hanrej* knockdown males loose post-mating behavior when they still have a higher number of sperm in their storage organs. However, they do not utilize this stored sperm for fertilization and accumulate mature oocytes in their ovaries. Such inability to detect and use sperm is a hallmark of defects in the SP network that mediates the binding of SP to sperm in the female RT [23,32]. In mates of *cornutus* or *hanrej* knockdown males, SP on sperm tails is not detectable at 4d after mating and is already markedly reduced 6h after mating when sperm storage is completed. This indicates that Cornutus and Hanrej are required for the transfer of SP onto sperm tails, either by directly interacting with sperm themselves or by involvement in preceding SP network signaling steps.

We establish that *cornutus*, though judged by single cell transcriptional analysis as a main cell marker gene with strong expression bias [35], is not required in this cell type for the female long-term post-mating response, but rather in secondary cells, whereas *hanrej* is required in main cells. For other members of the SP network that transiently bind to sperm together with SP, an origin from secondary cells (Lectin-46Ca, Lectin-46Cb) as well as from main cells (Antares, CG9997) has been implied before. The SP network thus appears to rely on a tight cooperation of secondary and main cell secretory products.

### Secondary cell DCG vesicle secretion as a key step for producing a functional SP network

Unusually large DCG vesicles are characteristic of secondary cells. Though surpassing the size of typical mammalian DCG vesicles in the nervous system and hormone producing glands by more than an order of magnitude, they share evolutionary conserved regulators of biogenesis and trafficking [36,54]. The large DGC vesicles not only contain and release material from DCGs, but also small intraluminal vesicles/exosomes [28] and electron dense filamentous structures [31, 59]. Mating induced DCG vesicle release stimulates their replenishment via an autocrine mechanism involving BMP signaling [55], and interference with this pathway has been previously shown to affect some aspects of female post-mating behavior and sperm handling, as well as the AG proteome [37]. In secondary cells of *iab-6*^*cocu*^ mutants, DCG vesicles are completely lost, along with other morphological changes such as cell shape and volume.

We find that loss of Cornutus specifically impairs the release of DCG vesicles during mating. In *cornutus* knockdown secondary cells, DCG vesicles still form, but are slightly more numerous and contain smaller condensed DCG cores in unmated males. This suggests that the final DCG maturation steps, aggregation of smaller minicores to a big central DCG within a vesicle [54] and triggered secretion of vesicles during copula require Cornutus, which itself localizes to DCG vesicles. Without Cornutus, the SP network does not function normally, and all canonical aspects of the long-term post-mating response are lost. Currently, it is not known if this SP network failure results from a lack of Cornutus transfer to the female RT or from the lack of other SP network proteins that are released by DGC vesicles as part of the DCGs, exosomes or filamentous structures. The main candidates for DCG vesicle release dependent SP network proteins are Lectin-46Ca and Lectin-46Cb.

More detailed studies of the interactions of previously known SP network members with the two newly identified factors, Cornutus and Hanrej, are needed to clarify which parts of the network depend directly on DCG vesicle secretion.

In conclusion, disrupted secondary cell secretory identity in *iab-6*^*cocu*^ mutant males influences the abundances of more than 10% of known SFP in the AG, some of which are likely to be subjected to altered translation, processing and secretion, stability and/or transfer. In the future, it will be of interest to investigate the mechanisms by which secondary and main cells interact and the interdependence of their secretory products in the seminal fluid in both male and female RT. Such interdependence is of functional relevance within the SP network, which relies on SFPs from main and secondary cells. In the *iab-6*^*cocu*^ mutant transferome, multiple members of the SP network are downregulated. The mechanism of this concerted regulation remains to be explored, as does the exact role of DCG vesicle products in mediating the long-term post-mating response.

## Materials and methods

### Drosophila culture, transgenic Drosophila stocks and Drosophila gene analysis

Flies were maintained on a standard medium containing sugar, oatmeal, cornmeal, agar, and yeast, under 12h light:12h dark cycle at 25°C. All experimental flies were collected after eclosion and aged 4–7 days. Males were individually aged in a flat bottom 1.5mL 96 well block filled with 0.5mL food per well and covered with a PCR foil with air holes. Females were aged in groups of 10–15 in food vials. Fly stocks used in this study are listed in S3 Table. The exact genotypes of experimental flies are listed in S4 Table. To generate *UAS-cornutus-Egfp* flies, the protein coding sequence with a C-terminal Egfp tag of cornutus was cloned into the *pUASTattB* vector and the construct was inserted by PhiC31 mediated integration into the *attp40* landing site.

We used FlyBase (release FB2026_02) find information on gene sequence, expression and transgenic stock availability [60,61].

### Mass Spectrometry

A male (for unmated sample) or a male and a virgin female (for mated samples) were transferred to a perforated 1.5 mL Eppendorf tube and kept under observation until the occurrence of copulation. Immediately after the end of copulation (for the mated group), flies were frozen in liquid nitrogen. Tissues were dissected with fine forceps in ice cold PBS, transferred to a 1.5mL Eppendorf tube and frozen in liquid nitrogen. Each sample contained tissues from five individual flies, and each condition was analyzed in five biological replicates. Male tissues were the paired accessory glands, removed from the ejaculatory duct at their bases. Female tissues were the reproductive tract with the ovaries removed at the base of the lateral oviducts. Tissues were lyophilized dry before being resuspended in 8M urea. As urea was added, the tissues were thoroughly macerated with the tip of the pipette. Samples were reduced for 60min in 10mM dithiothreitol, followed by alkylation for 60min in 30mM iodoacetamide. The urea concentration was lowered to <1 M before overnight digestion with trypsin (2 μg/sample) at 37°C. The samples were desalted on homemade columns packed with octadecyl C18 disks (Empore, 3M) and dissolved in 0.1% formic acid. The desalted samples were loaded onto a trap column (2 cm x 75μm inner diameter) and separated on an analytical column (15 cm x 75μm inner diameter) packed with 3 μm C18 beads (Dr. Maisch, GmbH). Samples were eluted over 60min and analyzed on an Orbitrap Eclipse Tribid mass spectrometer (Thermo Fisher Scientific). Samples were analyzed in technical triplicates and injected in alternating order. Data files were processed in Proteome Discoverer 2.4 with the Sequest HT search engine. The *Drosophila melanogaster* UniProt reference proteome (UP000000803) was used as a database, with the following parameters: 10 ppm precursor mass accuracy, 0.6 Da fragment mass accuracy, trypsin as digestion enzyme, maximum three missed cleavage sites, Oxidation (M) as variable modification and Carbamidomethyl (C) as fixed modification. Unique peptides were quantified using a label-free approach based on precursor intensity or area to compare protein abundances across samples. Protein abundances were normalized to total peptide amount.

### Mass spectrometry data analysis

Data were filtered to only include proteins identified with a 1% FDR at the peptide level and to only include proteins identified in at least 4 out of 5 biological replicates (in either condition). The median of the technical replicates was used for further analyses. Protein abundances were base 2 log transformed before further processing. Missing values were imputed using a probabilistic minimum method. A Gaussian distribution was fit to the log2 transformed abundances, downshifted by 1.8 standard deviations and normalized to have a 30% standard deviation, to simulate the detection threshold of the mass spectrometer. Missing values were then drawn at random from this distribution. The imputation was performed 1000 times, and only proteins significantly different in abundance in 60% of the imputations were used to specify differently abundant proteins. P-values were calculated using an unpaired equal variance two-sample t-test and FDR-corrected using the Benjamini-Hochberg procedure. Fold change was calculated as the mean of the pairwise fold changes between log2 abundances of *iab-6*^*cocu*^ and wild type samples. Proteins were considered differentially abundant at an absolute log2 fold change of ≥1 and an adjusted p-value< 0.05. Hierarchical clustering was performed using the MATLAB function clustergram, with average linkage and Euclidean distances on standardized protein abundances.

### Behavioral assays

Behavioral assays were conducted at 25°C, 60% humidity at circadian time 0–3hrs after lights on or 3–0hrs before lights off. Female copulation song was recorded and analyzed as previously described [62]. Mating assays and tests of post-mating receptivity were performed as previously described [63,64]. Female virgin flies were paired with experimental males for 1h in circular mating chambers with 10mm diameter, 4mm height. If mating occurred, females were collected and kept in groups of 5–10 flies in food vials for 4 days. Remating was tested with wild type males for 1h in mating chambers. For assessing egg laying, virgin females were individually mated in mating chambers and then individually housed in 5ml vials filled with 1ml of fly food. Every 24hrs, females were transferred to new vials and eggs laid were counted.

### Sperm and egg chamber counts

To quantify female sperm storage, the spermatheca and seminal receptacle of mated females were dissected in 4°C PBS and mounted in a drop of PBS on a glass slide by pressing down gently a coverslip onto the organs. ProtamineB-Egfp labeled sperm heads were imaged with a fluorescent microscope (Nikon Eclipse Ni, with 20x/0.5 and 40x/0.75 Plan Fluor Air Objectives) and counted using the Fiji/ImageJ cell counter plug-in. To quantify egg chambers, ovaries of mated females were dissected in 4°C PBS, fixed in 4% paraformaldehyde with 0.3% TritonX for 25min at room temperature, washed three times in PBS with 0.3% TritonX and mounted in Vectashield containing Dapi (Vector labs, RRID: AB_2336790). Ovaries were imaged with a Leica STELLARIS8 FALCON confocal microscope equipped with a HC PL APO 20x/0,75 multi-immersion objective. Egg chambers were staged based on nuclear staining [65].

### Immunohistochemistry

Immunohistochemistry of fly nervous systems and reproductive tracts was performed as described previously [66]. Tissues were dissected in 4°C PBS, fixed in 4% paraformaldehyde with 0.3% TritonX, stained with mouse nc82 antibody (Developmental Studies Hybridoma Bank, RRID:AB_2314866, for nervous systems), rabbit anti-GFP antibody (Torrey Pines Biolabs, RRID: AB_10013661) followed by respective secondary antibodies (Goat IgG (H + L) Alexa Fluor 488 and 647) and Alexa Fluor 647 conjugated Phalloidin (Thermo Fisher, RRID: AB_2620155, for reproductive tracts) and mounted in Vectashield (Vector labs, RRID: AB_2336789).

Immunohistochemistry of sperm-bound SP was performed as described previously [67]. Seminal receptacles of mated females were dissected on poly-L-lysine coated glass slides 4°C PBS, fixed in 4% paraformaldehyde for 15min at room temperature, washed three times in PBS, blocked for 30 minutes in 5% normal goat serum (NGS) and then incubated overnight at 4°C with rabbit anti-SP primary antibody (M. Wolfner lab, 1:200), followed by incubation for 2–3hrs at room temperature with secondary antibody (Goat IgG (H + L) Alexa Fluor 488). Samples were mounted in Vectashield (Vector labs, RRID: AB_2336789). Dissection and staining steps were carried out within a ring of double-sided adhesive tape attached to the glass slide to confine the sample area and prevent sample loss. Tissues and sperm bundles were imaged with a Leica STELLARIS8 FALCON microscope equipped with a HC PL APO 20x/0,75 multi-immersion objective.

### DCG imaging data analysis

For imaging of mfas-Egfp and Cornutus-Egfp, accessory glands were dissected in 4°C PBS and mounted between two coverslips in a drop of PBS, with a small amount of food-grade silicone grease serving as spacer between the coverslips. The coverslips were inserted in a metal frame and imaged with a Leica STELLARIS8 FALCON microscope (HC PL APO 100x/1,40 oil objective for assessing DCG size, HC PL APO 20x/0,75 multi-immersion objective for counting DCG). To assess the size of the DCG we used the plug-in “analyse particle” in FIJI software. For each genotype 4 animals with 3–6 secondary cells per accessory gland were evaluated.

To assess DCG number, they were counted based on manual inspection of z-stacks using merged fluorescence and transmitted light channels. Approximately 10 secondary cells per AG were analyzed. Only condensed DCGs were included in the analysis, characterized by sharply defined circular structures with a bright GFP signal. Mated flies were anesthetized on ice immediately after the end of copulation and their accessory glands were imaged within 2hrs. For permutation testing of DGC release, the differences between the mean number of DCG per cell (Δmean) before and after mating were calculated. All before mating datapoints for different genotypes and all after mating datapoints for different genotypes were permuted 100.000 times, the p-value reports the probability of the difference of the absolute Δmean for the knockdown genotype to the Δmeans of both control genotypes being ≥ the observed value (code deposited under: github.com/KinaUna/PhilipsbornPermutations).

## Supporting information

S1 Fig Copulation song of females mated to males depleted for candidate SFPs downregulated in the *iab-6*^*cocu*^ mutant transferome. Female copulation song pulses/ min copulation of wild type females mated to experimental males, in which candidate SFPs were depleted by RNAi expressed by the ubiquitous *tubulin-GAL4* driver. SFP names are shown above the graph, identifiers of the RNAi lines under the graph. Each datapoint represents one copulating female, with n indicated under the x axis. Error bars represent median and interquartile range, no significant difference was detected to the control (Mann-Whitney test, two sided).
(TIF)

S2 Fig Expression patterns of GAL4 driver lines to specifically manipulate main and secondary cells of the accessory gland. **A** Expression pattern of *dve-GAL4*, *antares-GAL4* and *dsx-GAL4* in the male reproductive tract, antibody staining against cd8-Egfp in GAL4 expressing cells is shown in green, phalloidin based actin staining in muscle is shown in magenta. The inset in the middle panel shows a single confocal imaging plane with non-labelled secondary cells indicated by white arrows. Scale bar, 100μm. **B** Expression strength of the GAL4 driver lines dve-GAL4, antares-GAL4 and dsx-GAL4 in main (main) and secondary (sec) cells of the accessory gland over 0–7d post eclosion in unmated males, represented by fluorescence intensity of unstained cd8-Egfp detected with fixed imaging settings (maximum intensity projections of full confocal stacks of AGs). Left rows: Egfp in green, transmitted light outlines of the reproductive tract in greyscale. White arrow indicates luminal Egfp labled membrane fragment. Right rows: Egfp fluorescence color code. Scale bar, 200μm. **C** Expression pattern of dve-GAL4 without (left) and with (right) suppression of neuronal expression by *nsyb-GAL80* in central nervous system (brain and vnc) and reproductive tract. Antibody staining against cd8-Egfp in GAL4 expressing neurons and gland cells is shown in green, anti-bruchpilot staining of the central nervous system neuropil in magenta, transmitted light outlines of the reproductive tract in greyscale. White arrows indicate peripheral nerves. Scale bar, 100μm. For **A-C**, a micrograph of one representative tissue (out of 5 or more samples per genotype with the same expression pattern) is shown. AG, accessory gland, SV, seminal vesicle, ED, ejaculatory duct, T, testis, sphi, sphincter region of AG. **D** Percentage of remating female flies after 4d when mated to males in which *cornutus* was depleted with RNAi expressed by the *dve-GAL4, nsyb-GAL80* driver. ****p < 0.0001, Fisher’s exact test.
(TIF)

S3 Fig Effect of Cornutus and Hanrej knockdown on remating at 2d and 3d and egg laying after cell specific knockdown. **A** Percentage of remating female flies after 2d and 3d when mated to males in which *cornutus* o*r hanrej* were depleted with RNAi expressed in different AG cell populations and to control males (right). ****p < 0.0001, ***p < 0.001, **p = 0.0011 Fisher’s exact test. n, number of females tested, is indicated under the x axis. **B** Number of eggs laid by females mated to males in which *cornutus* o*r hanrej* were depleted in main cells only (*antares-GAL4*) or in secondary cells only (*dsx-GAL4*), and to control males, over the course of six days following the mating. ****p < 0.0001, Kruskal Wallis test with Dunn’s multiple comparison.
(TIF)

S4 Fig Cornutus and Hanrej affect Sex Peptide binding to sperm at 2d and 3d after mating. **A** Immunohistochemistry of SP (anti SP) on sperm dissected from seminal receptacles and visualized in transmitted light 6h after the female’s mating to males in which *cornutus* o*r hanrej* were depleted with RNAi and control males, shown at high magnification. Scale bar, 5μm. **B** Immunohistochemistry of SP (anti SP) on sperm dissected from seminal receptacles and visualized in transmitted light 2d and 3d after the female’s mating to males in which *cornutus* o*r hanrej* were depleted with RNAi and control males. Scale bar, 20μm. A micrograph of one representative sperm bundle (out of 3–8 samples per condition) is shown. For full genotypes, see methods.
(TIF)

S5 Fig Localization of Cornutus-Egfp in DCG of males of different ages. Localization of Cornutus-Egfp, expressed with the *dve-GAL4* driver, in secondary cells DCGs. The distal part of a representative accessory gland is shown, with age and mating state of the male indicated. White arrowheads indicate small Egfp puncta in the main cells of 10d old males. Scale bar, 50μm.
(TIF)

S1 Table Detected proteins. The three sheets list detected proteins for 1) AG proteome non mated males (male nm), 2) AG proteome mated males (male m), and 3) RT proteome mated female (female m). Significantly up- or downregulated proteins in *iab-6*^*cocu*^ mutants or females mated to *iab-6*^*cocu*^ mutants are marked with “1” in column I and J, high-confidence SFPs are marked with “1” in column W. Columns K-O and P-T list protein abundancies of the 5 biological replicates (containing each tissues from 5 flies) for *iab-6*^*cocu*^ mutants (cocu) and wild type samples (wt).
(XLSX)

S2 Table Proteins detected in all three datasets. List of proteins detected in all three datasets with three groups of proteins that show significant abundance changes consistent with a downregulation in the male transferome (see Fig 1C).
(XLSX)

S3 Table Expression information and remating rates for SFPs downregulated in the *iab-6*^*cocu*^ mutant transferome. For downregulated proteins, mRNA expression levels according to the FlyAtlas 2 Anatomy RNA-Seq dataset (FlyBase release FB2026_02, Leader et al. 2018) are indicated, followed by RNAi lines used for screening for remating and remating rates. n gives the number of wild type females tested for remating with wild type males 4d after being mated to a knockdown male.
(DOCX)

S4 Table Drosophila stocks used. All genotypes refer to male flies. All female mates used in experiments were CS wild type. Chromosomes derived from CS wild type background are written “*+*”.
(DOCX)

S5 Table Genotypes of experimental flies. All genotypes refer to male flies. All female mates used in experiments were CS wild type. Chromosomes derived from CS wild type background are written “*+*”.
(DOCX)
