# Supplementary material for: Abdominal-B regulates the male seminal fluid transferome and new fecundity factors required for sperm sex peptide binding
Source: PLoS Genet. 2026 Sep 1;22(9):e1012298. doi: 10.1371/journal.pgen.1012298 (PMC13557496; doi:10.1371/journal.pgen.1012298)
Supplement: Supplementary file 1 [file pgen.1012298.s008.docx]

**S3 Table.**

**Expression information and remating rates for SFPs downregulated in the *iab-6^cocu^* mutant transferome.**

For downregulated proteins, mRNA expression levels according to the FlyAtlas 2 Anatomy RNA-Seq dataset (FlyBase release FB2026_02, Leader et al. 2018) are indicated, followed by RNAi lines used for screening for remating and remating rates. n gives the number of wild type females tested for remating with wild type males 4d after being mated to a knockdown male.

| **Gene** | **AG expression**  **(FlyAtlas 2)** | **Male expression outside AG (FlyAtlas 2)** | **RNAi line**  **(used with**  ***tub-GAL4*)** | **% Remating at 4 d** | **n** |
| --- | --- | --- | --- | --- | --- |
| *CG1701/*  *cornutus* | extremely high | Testis | VDRC_v106343 | 76 | 25 |
|  |  |  | VDRC_v29663 | 74 | 15 |
| *Ccp84Ad* | low | no | BDSC_62531 | 0 | 15 |
|  |  |  | BDSC_65912 | 0 | 11 |
| *Dup99B* | extremely high | Eye, Hindgut, Malphighian Tubule, Fat Body, Heart, Rectal Pad, Testis | BDSC_60069 | 0 | 14 |
| *CG11598* | moderately high | no-low | BDSC_64969 | 0 | 13 |
| *Spn75F* | extremely high | Fat Body, Testis | BDSC_51437 | 0 | 12 |
| *Obp51a* | extremely high | Hindgut, Fat Body, Rectal Pad, Testis | VDRC_v27143 | 7 | 14 |
|  |  |  | VDRC_v27144 | 0 | 22 |
| *CG31419* | very high | no-low | BDSC_57575 | 0 | 19 |
|  |  |  | BDSC_58243 | 0 | 18 |
| *CG31883* | extremely high | Hindgut, Fat Body, Testis | VDRC_v36488 | 0 | 21 |
|  |  |  | BDSC_65100 | 8 | 12 |
| *Lectin-46Ca* | extremely high | Fat Body, Testis | BDSC_56016 | 84 | 31 |
| *Sfp23F* | extremely high | Fat Body, Testis | VDRC_v330530 | 0 | 12 |
| *Lectin-46Cb* | very high | Fat Body | BDSC_67250 | 0 | 20 |
| *CG42564/*  *hanrej* | very high | Testis | BDSC_65910 | 74 | 19 |
|  |  |  | VDRC_v14158 | 86 | 14 |
| *CG9168* | very high | no-low | VDRC_v29065 | 11 | 28 |
|  |  |  | BDSC_56994 | 0 | 16 |
|  |  |  | BDSC_62976 | 0 | 19 |
| *CG31680* | extremely high | Fat Body, Testis | BDSC_58145 | 11 | 18 |
|  |  |  | VDRC_v50891 | 0 | 13 |
|  |  |  | VDRC_v50890 | 0 | 12 |
| *CG11600* | moderate | no-low | BDSC_66927 | 0 | 38 |
