# Supplementary material for: Abdominal-B regulates the male seminal fluid transferome and new fecundity factors required for sperm sex peptide binding
Source: PLoS Genet. 2026 Sep 1;22(9):e1012298. doi: 10.1371/journal.pgen.1012298 (PMC13557496; doi:10.1371/journal.pgen.1012298)
Supplement: Supplementary file 2 [file pgen.1012298.s009.docx]

**S4 Table.**

**Drosophila stocks used.**

| **Short name, label** | **Genotype** | **Identifier/RRID** |
| --- | --- | --- |
| wt, CS | Canton S wild type strain | Laboratory of B. Dickson |
| *iab-6 ^cocu^* | *+; +; iab-6 ^cocu^*  Mutant in wt background | Laboratory of F. Karch, R. Maeda, FBal0284736 |
| *tub-GAL4* | *y, w; P{tubP-GAL4}LL7* | BDSC_5138 |
| *dve-GAL4* | *w;;**P{dve-GAL4.13}30A* | Laboratory of H. Nakagoshi, FBti0152837 |
| *antares-GAL4* | *y, w; TI{CRIMIC.TG4.2}antr^CR00860-TG4.2^* | FBal0341147 |
| *dsx-GAL4* | *w;;TI{GAL4}dsx[GAL4]/TM6B, Tb* | BDSC_66674 |
| *nsyb-GAL80* | *w; P{w[+mC]=nSyb-GAL80.S}3* | BDSC_92154 |
| *protB-eGFP* | *+; P{protamineB-eGFP}2* | Derived from BDSC_58406 |
| *SP mutant* | *SP0(0325)* | BDSC_94694 |
| *SP deficiency* | *Df(3L)Δ130* | Laboratory of J.C. Billeter, FBab0029476 |
| *UAS-Dcr2* | *w, P{UAS-dicer2, w[+]}* | VDRC Id 60007 |
| *RNAi: CG1701, cornutus-IR, 1* | *w; P{KK109689}VIE-260B* | VDRC Id 106343 |
| *RNAi: CG1701, cornutus-IR, 2* | *w; P{GD15054}v29663* | VDRC Id 29663 |
| *RNAi: CG42564, hanrej-IR, 1* | *y, sc, v, sev; P{TRiP.HMC06173}attP40* | BDSC_65910 |
| *RNAi: CG42564, hanrej-IR, 2* | *w;; P{GD6109}v14158* | VDRC Id 14158 |
| *mfas-Egfp* | *y, w; Mi{PT-GFSTF.2}mfas[MI11275-GFSTF.2]* | BDSC_63204 |
| *UAS-cornutus-Egfp* | *w; P{UAS-cornutus-Egfp}attp40* | This study |
| *UAS-cd8-Egfp* | *w; P{UAS-cd8-Egfp}* | Laboratory of B. Dickson |
| *RNAi: Ccp84Ad-IR, 1* | *y, v; P{TRiP.HMJ24075}attP40/CyO* | BDSC_62531 |
| *RNAi: Ccp84Ad-IR, 2* | *y, sc, v, sev; P{TRiP.HMC06176}attP40* | BDSC_65912 |
| *RNAi: Dup99B-IR* | *y, sc, v, sev; P{TRiP.HMC05062}attP40* | BDSC_60069 |
| *RNAi: CG11598-IR* | *y, sc, v, sev; P{TRiP.HMC05843}attP40* | BDSC_64969 |
| *RNAi: Spn75F-IR* | *y, v; P{TRiP.HMC03151}attP2* | BDSC_51437 |
| *RNAi: Obp51a-IR, 1* | *w; P{GD6816}v27143* | VDRC Id 27143 |
| *RNAi: Obp51a-IR, 2* | *w; P{GD6816}v27144* | VDRC Id 27144 |
| *RNAi: CG31419-IR, 1* | *y, sc, v, sev; P{TRiP.HMC04584}attP40* | BDSC_57575 |
| *RNAi: CG31419-IR, 2* | *y, v; P{TRiP.HMJ22264}attP40/CyO* | BDSC_58243 |
| *RNAi: CG31883-IR, 1* | *y, sc, v, sev; P{TRiP.HMC05996}attP40* | BDSC_65100 |
| *RNAi: CG31883-IR, 2* | *w; P{GD14757}v36488* | VDRC Id 36488 |
| *RNAi: lectin-46Ca-IR* | *y, sc, v, sev; P{TRiP.HMC04312}attP40* | BDSC_56016 |
| *RNAi: Sfp23F-IR* | *w; P{VSH330530}attP40* | VDRC Id 330530 |
| *RNAi: lectin-46Cb-IR* | *y, sc, v, sev; P{TRiP.HMC06352}attP40* | BDSC_67250 |
| *RNAi: CG9168-IR, 1* | *w; P{GD14142}v29065* | VDRC Id 29065 |
| *RNAi: CG9168-IR, 2* | *y, sc, v, sev; P{TRiP.HMC04436}attP40* | BDSC_56994 |
| *RNAi: CG9168-IR, 3* | *y, v; P{TRiP.HMJ30053}attP40* | BDSC_62976 |
| *RNAi: CG31680-IR, 1* | *y, v; P{TRiP.HMJ22095}attP40* | BDSC_58145 |
| *RNAi: CG31680-IR, 2* | *w; P{GD17288}v50891* | VDRC Id 50891 |
| *RNAi: CG31680-IR, 3* | *w; P{GD17288}v50890* | VDRC Id 50890 |
| *RNAi: CG11600-IR* | *y, sc, v, sev; P{TRiP.HMS05393}attP40* | BDSC_66927 |
