# Supplementary material for: Abdominal-B regulates the male seminal fluid transferome and new fecundity factors required for sperm sex peptide binding
Source: PLoS Genet. 2026 Sep 1;22(9):e1012298. doi: 10.1371/journal.pgen.1012298 (PMC13557496; doi:10.1371/journal.pgen.1012298)
Supplement: Supplementary file 3 [file pgen.1012298.s010.docx]

**S5 Table.**

**Genotypes of experimental flies.**

All genotypes refer to male flies. All female mates used in experiments were CS wild type. Chromosomes derived from CS wild type background are written “*+*”.

| **Figure** | **label** | **Full genotype** |
| --- | --- | --- |
| 2A, S3 | *dve-GAL4* | *w, UAS-Dcr2; +/+; dve-GAL4/+* |
|  | *dve-GAL4, cornutus-IR, 1* | *w, UAS-Dcr2; P{KK109689}VIE-260B/+; dve-GAL4/+* |
|  | *dve-GAL4, cornutus-IR, 2* | *w, UAS-Dcr2; P{GD15054}v29663/+; dve-GAL4/+* |
|  | *dve-GAL4, hanrej-IR, 1* | *w; P{TRiP.HMC06173}attP40/+; dve-GAL4/+* |
|  | *dve-GAL4, hanrej-IR, 2* | *w, UAS-Dcr2; +/+; dve-GAL4/P{GD6109}v14158* |
|  | *antares-GAL4* | *w, UAS-Dcr2; antares-GAL4/+;+/+* |
|  | *antares-GAL4, cornutus-IR, 1* | *w, UAS-Dcr2; antares-GAL4/P{KK109689}VIE-260B; +/+* |
|  | *antares-GAL4, cornutus-IR, 2* | *w-, UAS-Dcr2; antares-GAL4/P{GD15054}v29663; +/+* |
|  | *antares-GAL4, hanrej-IR, 1* | *+; antares-GAL4/P{TRiP.HMC06173}attP40/+; +/+* |
|  | *antares-GAL4, hanrej-IR, 2* | *w, UAS-Dcr2; +/+; dve-GAL4/P{GD6109}v14158* |
|  | *dsx-GAL4* | *w, UAS-Dcr2; +/+; dsx-GAL4/+* |
|  | *dsx-GAL4, cornutus-IR, 1* | *w, UAS-Dcr2; P{KK109689}VIE-260B/+; dsx-GAL4/+* |
|  | *dsx-GAL4, cornutus-IR, 2* | *w, UAS-Dcr2; P{GD15054}v29663/+; dsx-GAL4/+* |
|  | *dsx-GAL4, hanrej-IR, 1* | *w; P{TRiP.HMC06173}attP40/+; dsx-GAL4/+* |
|  | *dsx-GAL4, hanrej-IR, 2* | *w, UAS-Dcr2; +/+; dsx-GAL4/P{GD6109}v14158* |
|  | *cornutus-IR, 1* | *+; P{KK109689}VIE-260B/+; +/+* |
|  | *cornutus-IR, 2* | *+; P{GD15054}v29663/+; +/+* |
|  | *hanrej-IR, 1* | *+; P{TRiP.HMC06173}attP40/+; +/+* |
|  | *hanrej-IR, 2* | *+; +/+; P{GD6109}v14158/+* |
| 2C, E | *dve-GAL4* | *w, UAS-Dcr2; +/+; dve-GAL4/+* |
|  | *cornutus-IR* | *+; P{KK109689}VIE-260B/+; +/+* |
|  | *dve>cornutus-IR* | *w, UAS-Dcr2; P{KK109689}VIE-260B/+; dve-GAL4/+* |
|  | *hanrej-IR* | *+; +/+; P{GD6109}v14158/+* |
|  | *dve>hanrej-IR* | *w, UAS-Dcr2; +/+; dve-GAL4/P{GD6109}v14158* |
| 2D | *dve-GAL4, protB-Egfp* | *w, UAS-Dcr2; protamineB-Egfp/+; dve-GAL4/+* |
|  | *dve-GAL4, protB-Egfp, cornutus RNAi* | *w, UAS-Dcr2; P{KK109689}VIE-260B/ protamineB-Egfp; dve-GAL4/+* |
|  | *dve-GAL4, protB-Egfp, hanrej RNAi* | *w, UAS-Dcr2; protamineB-Egfp/+; dve-GAL4/P{GD6109}v14158* |
| 3, S4 | *dve-GAL4* | *w, UAS-Dcr2; +/+; dve-GAL4/+* |
|  | *cornutus-IR* | *+; P{KK109689}VIE-260B/+; +/+* |
|  | *dve>cornutus-IR* | *w, UAS-Dcr2; P{KK109689}VIE-260B/+; dve-GAL4/+* |
|  | *hanrej-IR* | *+; P{TRiP.HMC06173}attP40/+; +/+* |
|  | *dve>hanrej-IR* | *w, UAS-Dcr2; P{TRiP.HMC06173}attP40/+; dve-GAL4/+* |
| 3 | *SP mutant* | *+; +/+; SP0(0325)/ Df(3L)Δ130* |
| 4A, B, C | *dve-GAL4* | *w, UAS-Dcr2; +/+; dve-GAL4/mfas-Egfp* |
|  | *cornutus-IR* | *+; P{KK109689}VIE-260B/+; +/+* |
|  | *dve>cornutus-IR* | *w, UAS-Dcr2; P{KK109689}VIE-260B/+; dve-GAL4/ mfas-Egfp* |
|  | *hanrej-IR* | *+; P{TRiP.HMC06173}attP40/+; +/+* |
|  | *dve>hanrej-IR* | *w, UAS-Dcr2; P{TRiP.HMC06173}attP40/+; dve-GAL4/+* |
| 4D, S5 | *dve>cornutus-Egfp* | *w; P{UAS-cornutus-Egfp}attp40/+; dve-GAL4* |
| S1 | control | *+; +/+; tub-GAL4/+* |
|  | CG1701, VDRC_106343 | *+; P{KK109689}VIE-260B/+; tub-GAL4/+* |
|  | CG1701, VDRC_29663 | *+; P{GD15054}v29663/+; tub-GAL4/+* |
|  | Ccp84Ad, BDSC_62531 | *+; P{TRiP.HMJ24075}attP40/+; tub-GAL4/+* |
|  | Ccp84Ad, BDSC_65912 | *+; P{TRiP.HMC06176}attP40/+; tub-GAL4/+* |
|  | Dup99B, BDSC_60069 | *+; P{TRiP.HMC05062}attP40/+; tub-GAL4/+* |
|  | CG11598, BDSC_64969 | *+; P{TRiP.HMC05843}attP40/+; tub-GAL4/+* |
|  | Spn75F, BDSC_51437 | *+; +/+; tub-GAL4/P{TRiP.HMC03151}attP2* |
|  | Obp51a, VDRC_27143 | *+; P{GD6816}v27143/+; tub-GAL4/+* |
|  | Obp51a, VDRC_27144 | *+; P{GD6816}v27144/+; tub-GAL4/+* |
|  | CG31419, BDSC_57575 | *+; P{TRiP.HMC04584}attP40/+; tub-GAL4/+* |
|  | CG31419, BDSC_58243 | *+; P{TRiP.HMJ22264}attP40/+; tub-GAL4/+* |
|  | CG31883, BDSC_65100 | *+; P{TRiP.HMC05996}attP40/+; tub-GAL4/+* |
|  | CG31883, VDRC_36488 | *+; +/+; P{GD14757}v36488/ tub-GAL4* |
|  | lectin-46Ca, BDSC_56016 | *+; P{TRiP.HMC04312}attP40/+; tub-GAL4/+* |
|  | Sfp23F, VDRC_330530 | *+; P{VSH330530}attP40/+; tub-GAL4/+* |
|  | lectin-46Cb, BDSC_67250 | *+; P{TRiP.HMC06352}attP40/+; tub-GAL4/+* |
|  | CG9168, VDRC_29065 | *+; P{GD14142}v29065/+; tub-GAL4/+* |
|  | CG42564, VDRC_14158 | *+; +/+; P{GD6109}v14158/ tub-GAL4* |
|  | CG9168, BDSC_56994 | *+; P{TRiP.HMC04436}attP40/+; tub-GAL4/+* |
|  | CG9168, BDSC_62976 | *+; P{TRiP.HMJ30053}attP40/+; tub-GAL4/+* |
|  | CG31680, BDSC_58145 | *+; P{TRiP.HMJ22095}attP40/+; tub-GAL4/+* |
|  | CG31680, VDRC_50891 | *+; +/+; P{GD17288}v50891/tub-GAL4* |
|  | CG31680, VDRC_50890 | *+; +/+; P{GD17288}v50890/tub-GAL4* |
|  | CG11600, BDSC_66927 | *+; P{TRiP.HMS05393}attP40/+; tub-GAL4/+* |
| S2A, B, C | *dve-GAL4* | *w; UAS-cd8-Egfp/+; dve-GAL4/+* |
|  | *antares-GAL4* | *w; UAS-cd8-Egfp/antares-GAL4; +/+* |
|  | *dsx-GAL4* | *w; UAS-cd8-Egfp/+; dsx-GAL4/+* |
| S2C | *dve-GAL4, nsyb-GAL80* | *w; UAS-cd8-Egfp/+; dve-GAL4, nsyb-GAL80/+* |
| S2D | *dve-GAL4, nsyb-GAL80* | *w, UAS-Dcr2; +/+; dve-GAL4, nsyb-GAL80/+* |
|  | *dve-GAL4, nsyb-GAL80, cornutus-IR, 1* | *w; P{GD15054}v29663/+; dve-GAL4, nsyb-GAL80/+* |
|  | *dve-GAL4, nsyb-GAL80, cornutus-IR, 2* | *w; P{KK109689}VIE-260B/+; dve-GAL4, nsyb-GAL80/+* |
